# Case report about the management of a late Gastro-Gastric Fistula after Laparoscopic Gastric Bypass, with the finding of an unexpected foreign body

**DOI:** 10.1016/j.ijscr.2020.01.012

**Published:** 2020-01-23

**Authors:** Simon Rizk, Wissam El Hajj Moussa, Nidal Assaker, Elias Makhoul, Elie Chelala

**Affiliations:** a Department of Gastroenterology, University Hospital Notre Dame des Secours, Byblos-Lebanon affiliated to Faculty of Medicine and Medical Sciences of the Holy Spirit University of Kaslik (USEK), Jounieh, Lebanon; b Department of General Surgery, University Hospital Notre Dame des Secours, Byblos-Lebanon affiliated to Faculty of Medicine and Medical Sciences of the Holy Spirit University of Kaslik (USEK), Jounieh, Lebanon

**Keywords:** Gastro-Gastric Fistula, Faucher tube stapling, Revisional gastric bypass, Laparoscopy, Gastric dysplasia, Sub-total gastrectomy

## Abstract

**Background:**

Gastro-Gastric Fistula is a rare but potentially serious complication of Roux-en-Y Gastric Bypass. Orogastric tube stapling is an adverse bariatric surgery iatrogenic complication that surgeons should be aware of and that has rarely been described.

**Clinical case:**

A 51-year-old patient, operated in our University Hospital Center of a Gastric Bypass 3 years ago, presented on consultation with anemia and weight regain (BMI 36). An upper Gastrointestinal (GI) endoscopy showed a Gastro-Gastric Fistula and visualized the tip of a Faucher tube fixed in the blind pouch and an erosive ulceration on the gastrojejunal anastomosis. Multiple biopsies showed a low-grade dysplasia in the remnant stomach. A subtotal gastrectomy was performed with refashioning of the gastrojejunal anastomosis.

**Discussion:**

Anemia and weight regain, with or without the association of marginal ulcers are the most common signs of Gastro-Gastric Fistula after Gastric Bypass (1–6%). Surgical treatment remains the standard of care and should be tailored to the size and location of the fistula and the status of the gastrojejunal anastomosis. Orogastric tube perioperative complications are rare occurrences during bariatric surgery and not reported at a later stage. They can be associated with significant morbidity. Prevention strategies must be taken and standardized to prevent such events.

**Conclusion:**

The surgical option remains the standard of treatment and can be performed safely in Gastro-Gastric Fistula Type II. Orogastric tube accidental complications should be identified preferably perioperatively and measures of prevention should be implied to avoid such events.

## Highlights

• Anemia & Weight Regain are possible signs of Gastrogastric fistula post RYGB.
• Preventive Gastrectomy is indicated in presence of Dysplasia in the Gastric Remnant.
• Orogastric Tube Stapling can be immediately detected or discovered years after RYGB.
• A series of protocols should be respected to avoid Orogastric Tube Stapling.

## 1 Introduction

The work has been reported in line with the SCARE criteria [1].

Driven by the increase in the epidemic of obesity worldwide, bariatric surgery has gained popularity in the last few decades. Bariatric surgery is considered the front line of morbid obesity treatment, with Gastric Bypass gaining popularity and still being considered as the gold standard. Gastro-Gastric Fistula is a known rare complication after Gastric Bypass surgery, opening an abnormal communication between the excluded gastric remnant and the neo gastric pouch. It can lead to weight regain, marginal ulcers and epigastric pain. The incidence varies from 1 to 6% [[2], [3], [4], [5], [6]].

Laparoscopic Roux-en-Y Gastric Bypass (LRYGB) remains a technically demanding surgery and orogastric tube stapling can occur during the performance of the gastrojejunal anastomosis. It is a rare yet dreadful complication in bariatric surgery.

Malignancy in the gastric remnant is very rare following gastric bypass surgeries. However, endoscopic evaluation and follow up remain very difficult due to the isolated nature of the pouch indicating that a dysplasia should not be ignored.

## 2 Case report

A 51-year-old patient, with a history of an efficient RYGB performed 3 years ago, presented with dizziness, light headedness and weight regain. Lab tests showed anemia with Hemoglobin 8.8 g/dl, iron deficiency, and controlled diabetes by HbA1c 6.4% (11.4% prior to LRYGB).

**Fig. 1:**
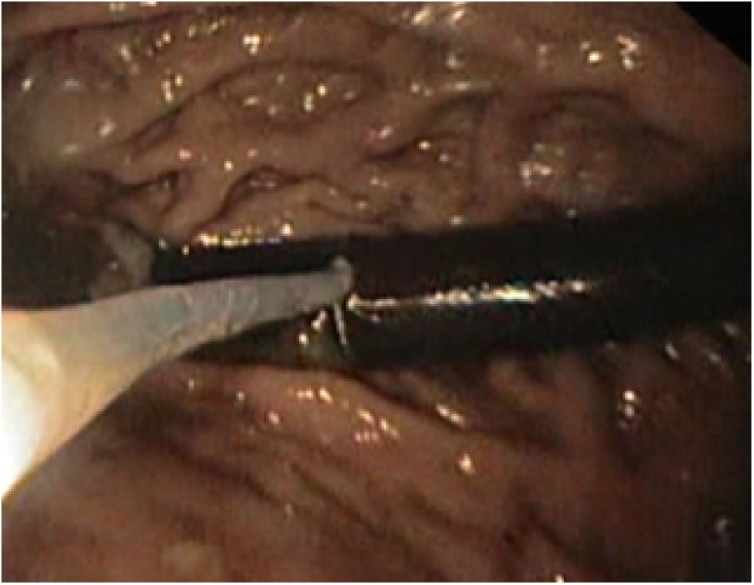
Fixed Faucher Tube in the Gastric Remnant.

An upper GI endoscopy was performed showing a large Gastro-Gastric Fistula between the upper Capella pouch and the gastric remnant. An erosive ulceration was also found on the gastrojejunal anastomosis. Multiples biopsies were taken, showing metaplastic intestinal cells with a low-grade dysplasia, in the gastric remnant. Finally, the tip of a Faucher tube was found fixed in the blind pouch distally and could not be retrieved endoscopically (Fig. 1). Retrospectively, the written report of the gastrotraphin® swallow that was performed on the 1st day post-op showed a normal passage, no sign of leakage and no foreign body was present in the gastric remnant.

**Fig. 2:**
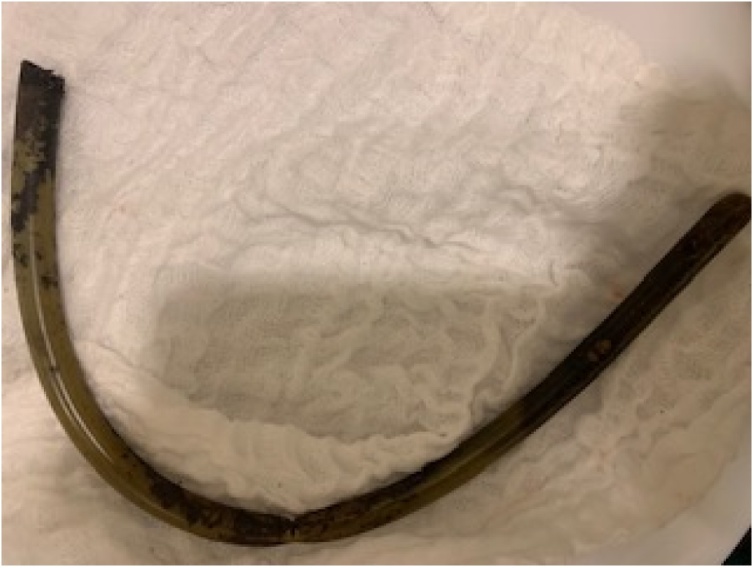
Faucher Tube extracted from gastric remnant.

Following both diagnoses, the patient underwent a laparoscopic subtotal gastrectomy with refashioning of the gastrojejunal anastomosis. The gastric remnant was resected completely, including the Faucher Tube of 20 cm, along the greater curvature (Fig. 2).

The two days follow-up course was uneventful. Gastrotraphin® swallow on day 1 post-op, was normal. Anatomic pathology results showed inflammatory reactivity surrounding the Gastric Fistula site, in the absence of any residual signs of dysplasia or malignancy. The patient was seen at 6 months post-op without anemia, in good health, and with a 15 kg weight-loss.

## 3 Discussion

Gastro-Gastric Fistula is a rare but known complication of RYGB, resulting from a communication between the Capella pouch and the excluded stomach [7]. The incidence rate described in the literature is believed to be underestimated due to lack of follow up [[2], [3], [4], [5], [6]]. Patients with Gastro-Gastric Fistula may be asymptomatic and may present with nonspecific symptoms such as weight regain, epigastric pain, nausea, vomiting, sometimes anemia as in our case, and even bleeding [[2], [3], [4], [5]]. An upper endoscopy and upper GI contrast study should be performed to confirm diagnosis [8]. Management of Gastro-Gastric Fistulas depends on the symptoms, size, location and its classification (according to Ribeiro-Parenti et al. type I or II) [9].

Type I: More than 1 cm above the gastrojejunal anastomosis

Type II: Less than 1 cm above the gastrojejunal anastomosis

In the case of type II or the presence of intractable marginal ulcers, the anastomosis should be resected and recreated [3,5,6,10].

In type I, conservative medical treatment consists of high PPI doses and should be started for symptomatic patients without weight regain. Small fistulas might close spontaneously on medical treatment alone [11]. Eventually, endoscopic repair can be safely used and shows great results for fistulas <10 mm in diameter. A variety of methods can be used including endoclips, endosutures, and stents [10,11].

In type II, surgical treatment remains the standard of care for large fistulas and those that fail conservative or endoscopic treatment. Multiple surgical options can be performed depending on the anatomical findings and the surgeon’s preference. A simple fistulectomy could be eventually performed. However, due to the fear of marginal ulceration and increased risk of recurrence, a more radical remnant gastrectomy is recommended, with 87–100% symptom resolution [12,13].

Orogastric tube stapling is a seldomly reported complication of bariatric surgery, with an incidence of 0.5–1.2%, more often detected perioperatively with an immediate repair. These occurrences might be increased in motorized staplers due to less tactile perception.

However, very late stage, years after surgery as in our case, complications have rarely been reported in the literature and hence should be emphasized as they may lead to severe complications if unrecognized [14].

Early perioperative recognition and repair of these complications is very important to reduce morbidity, and prevention strategies should be implemented to avoid them. Active communication with anesthetists is imperative to ensure the mobility of the orogastric tube during stapling and complete removal by checking the tip integrity of the Faucher tube. There are many intraoperative cues a surgeon should be aware of to avoid these complications: failure of a stapler to fire correctly, deformity of the stapler jaws, the need to use excessive force to close a stapler and finally control of the integrity of the Faucher tube, when removed [15].

Malignancy post RYGB is a rare occurrence with only around 30 cases reported in the literature [16]. Detecting it is a real challenge due to the difficulty in reaching the blind gastric remnant endoscopically and the vague nature of the symptoms. Gastric mucosal dysplasia is usually treated with endoscopic resection and reevaluation with regular follow ups [17] prior to surgery. However, in this case, given the exceptional anatomy and the extreme difficulty to access the blind gastric remnant, the decision was made to perform a subtotal gastrectomy. Two cases were reported with a gastric carcinoma in situ and both were treated with a remnant gastrectomy [17].

In our case report, the final biopsies didn’t find residual dysplasia on the Gastrojejunal site. A protective and selectively subtotal gastrectomy was performed anyway to eliminate both fistula recurrence and increased dysplasia.

## 4 Conclusion

The Remnant Gastrectomy, associated with pouch refashioning, remains the standard treatment and can be performed safely in Gastro-Gastric Fistula, type II. Orogastric tube accidental complications should be preferably identified perioperatively. Strict measures of prevention protocols and control should be implied to avoid such events. Finally, the surgeon should keep a high awareness for dysplasia and malignancies prior to bariatric surgery in the excluded stomach, which should be treated aggressively due to the difficulty of reaching these lesions endoscopically.

## Sources of funding

All authors declare having received no funding for the present case report.

## Ethical approval

The study is exempt from ethnical approval in our institution.

## Consent

Written informed consent was obtained from the patient for publication of this case report and accompanying images. A copy of the written consent is available for review by the Editor-in-Chief of this journal on request.

## Author’s contribution

The study conception of the case report, the supervision and finalisation were executed by the corresponding author and senior surgeon Pr Elie Chelala.

The first author, Simon Rizk PGY4 general surgery, wrote the draft paper in coordination for the review of the literature with Nidal Assaker PGY4 and Wissam El Hajj Moussa PGY5.

Elias Makhoul, Senior gastroenterologist, performed the gastroscopy and contributed in the decision making, management and supervision of the work.

## Registration of research studies

This is not a study of a series that needs a research registration, and therefore a case report doesn’t need it.

## Guarantor

Pr Elie Chelala is the coordinator and corresponding author.

## Provenance and peer review

Not commissioned, externally peer-reviewed.

## Declaration of Competing Interest

All authors declare having no conflict of interest in the current case report.
